# Acute dental infections managed in an outpatient parenteral antibiotic program setting: prospective analysis and public health implications

**DOI:** 10.1186/s12879-017-2303-2

**Published:** 2017-03-09

**Authors:** William J. Connors, Heidi H. Rabie, Rafael L. Figueiredo, Donna L. Holton, Michael D. Parkins

**Affiliations:** 1 Department of Medicine, University of Calgary, Foothills Medical Centre, 1403, 29th Street NW, Room 303, 3rd Floor North Tower, Calgary, AB T2N 2T9 Canada; 2 Dental Public Health Clinics, Alberta Health Services, Chumir Dental Clinic, 6th Floor, 1213 4th Street SW, Calgary, AB T2R 0X7 Canada; 3 Population, Public and Aboriginal Health, Alberta Health Services, Coronation Plaza 104, 14310 – 111 Avenue, Edmonton, AB T5M 3Z7 Canada; 4 Department of Microbiology, Immunology and Infectious Diseases, University of Calgary, Health Sciences Centre, 3330 Hospital Drive NW, Calgary, AB T2N 4N1 Canada; 5 Division of Infectious Diseases, Department of Medicine, Clinical Lecturer - University of Calgary, Foothills Medical Centre, 1403 29th Street NW, Room 303, 3rd Floor North Tower, Calgary, AB T2N 2T9 Canada

**Keywords:** Periapical abscess, Antibiotics, Healthcare costs, Parenteral infusions, Public health dentistry, Odontogenic infection

## Abstract

**Background:**

The number of Acute Dental Infections (ADI) presenting for emergency department (ED) care are steadily increasing. Outpatient Parenteral Antibiotic Therapy (OPAT) programs are increasingly utilized as an alternative cost-effective approach to the management of serious infectious diseases but their role in the management of severe ADI is not established. This study aims to address this knowledge gap through evaluation of ADI referrals to a regional OPAT program in a large Canadian center.

**Methods:**

All adult ED and OPAT program ADI referrals from four acute care adult hospitals in Calgary, Alberta, were quantified using ICD diagnosis codes in a regional reporting system. Citywide OPAT program referrals were prospectively enrolled over a five-month period from February to June 2014. Participants completed a questionnaire and OPAT medical records were reviewed upon completion of care.

**Results:**

Of 704 adults presenting to acute care facilities with dental infections during the study period 343 (49%) were referred to OPAT for ADI treatment and 110 were included in the study. Participant mean age was 44 years, 55% were women, and a majority of participants had dental insurance (65%), had seen a dentist in the past six months (65%) and reported prior dental infections (77%), 36% reporting the current ADI as a recurrence. Median length of parenteral antibiotic therapy was 3 days, average total course of antibiotics was 15-days, with a cumulative 1326 antibiotic days over the study period. There was no difference in total duration of antibiotics between broad and narrow spectrum regimes. Conservative cost estimate of OPAT care was $120,096, a cost savings of $597,434 (83%) compared with hospitalization.

**Conclusions:**

ADI represent a common preventable cause of recurrent morbidity. Although OPAT programs may offer short-term cost savings compared with hospitalization, risks associated with extended antibiotic exposures and delayed definitive dental management must also be gauged.

**Electronic supplementary material:**

The online version of this article (doi:10.1186/s12879-017-2303-2) contains supplementary material, which is available to authorized users.

## Background

Despite substantial advances in dental public health over the past century, recent trends in the United States and Canada reveal increasing rates of emergency department (ED) visits and hospitalizations for acute dental infections (ADI) [1–4]. A parallel trend has been documented in Europe with Germany, Britain and Finland showing similarly increased ED visits for ADI [5–7]. While retrospective studies in North America and Europe have characterized this epidemic and estimated associated health system resource and financial costs, little is known about the individual and community consequences resulting from acute medical management, particularly for the vast majority of patients with ADI who are not hospitalized.

Between 2000 and 2010 non-traumatic dental visits to EDs in the United States increased from 1.1 to 2.1 million, accounting for an increase in the proportion of all emergency visits from 1 to 1.5% [8]. Estimates from a combination of census and provincial reporting data in Canada reveal that 1.8 million people, representing 5.4% of the total population, reported one or more dental visits to EDs over their lifetime [4]. Of non-traumatic dental visits to EDs in the United States and Canada it is estimated that dental infections represent 40–60% of cases [4, 8]. ADIs represent a severe form of dental infection potentially resulting in systemic complications. Definitive management of ADI requires dental intervention; generally tooth extraction or root canal treatment, and often hospitalization. Antibiotics may serve a limited temporizing role pre- and peri-intervention but are not definitive management alone [9, 10]. Between 2000 and 2008 ADI hospitalization in the United States increased by 41%, after adjustment for growth, resulting in 8191 admissions in 2008 with attributed healthcare costs exceeding $105 million [2, 11].

Social determinants of health and related dental health policy have been shown to play an important role in the epidemiology of dental infections. Rates of dental visits to EDs and hospitalization for ADI are highest amongst individuals who are uninsured and poor [2, 4, 8, 12]. It is estimated that a quarter of Americans and over a third of Canadians do not have dental insurance, the proportion uninsured increasing to more than half amongst those in lower socio-economic groups [13, 14]. Outside of childhood and a limited number of special populations programs, routine dental health is not publicly funded in the United States or Canada [14–16]. In Canada, hospital and physicians’ services are publicly funded. However, only about 6% of all dental expenditures are publicly funded [17]. As a result of this lack of coverage and the division of dental and medical health, dental infections, although preventable, often progress ignored by healthcare policy until advanced and threatening overall medical health.

The vast majority of individuals with non-severe ADI in Canada never present to the EDs, rather they attend their dentist or general practitioner for urgent management that may entail definitive surgical intervention and/or temporizing oral antibiotics. Generally it is only severe cases with refractory pain, significant secondary facial cellulitis, or limiting trismus that are either referred to EDs or self attend. Whereas the majority of severe ADI have historically been managed with hospitalization, the modern expansion of Out-patient Parenteral Antibiotic Therapy programs (OPAT) have allowed for many severe ADI presenting to EDs to now be managed in the out-patient setting [18].

A detailed overview of OPAT programs has recently been published elsewhere [19]. Using this model, in Calgary, Alberta, urgent and emergency medical care facilities increasingly refer severe ADI that are medically fit for outpatient management to a regional OPAT program administered through each of the four adult acute care hospitals [20]. This comprehensive program now receives over 300 ADI referrals per year. As antibiotic therapy is only a bridge to a permanent solution for the dental problem, the OPAT program therefore serve as a point of referral to dental services. Despite the prevalence of patients with dental disease seeking acute medical care, to our knowledge there has not been any published research investigating individuals’ characteristics and the management of ADI through OPAT programs. To address this information gap, the objectives of this study were to prospectively assess the socio-medical profile and clinical management of patients treated for ADI in Calgary’s OPAT program, and to identify healthcare cost difference between hospital and OPAT management.

## Methods

This study was prospectively conducted in Calgary, Alberta, between February 1^st^ and June 30^th^, 2014. Designed as a quality improvement study, formal sample size calculation was not performed. A convenience sample was used with target sample size informed by the OPAT referral volumes of the preceding year (average 30 ADI referrals per month). The present study aimed to enrol 24 participants for each of the five months (*N* = 120), targeting an estimated 80% enrolment of all OPAT ADI referrals so as to provide a representative sample. The local health ethics board approved this study (REB-13-0935).

In our health zone, four EDs and two urgent care facilities provide all acute medical care to the metropolitan area with a population of approximately 1.2 million and serve as the ADI referral base (thus representing population based data). Participant enrollment and data collection took place in the OPAT program, consisting of four separate sites operating at each of the acute care facilities. The Calgary OPAT program provides community-based parenteral antibiotic therapy seven days a week under the guidance of an infectious diseases physician for indicated infections following referral. Initial OPAT assessments occurs within 24 h of referral and patients are discharged from the program when parenteral antibiotics are no longer needed, with therapy either transitioned to oral formulation or discontinued.

Eligible participants for this study were adults, 18 years of age and older, referred to OPAT for ADI management. Patients were excluded if they did not reside in the Calgary Health Zone to allow population level assessment. For the purpose of this study ADI was defined as new onset or acute worsening dental focus infection requiring parenteral antibiotic therapy based upon physician assessment. Preliminary determination of the need for parenteral antibiotics in the OPAT setting was made by the referring ED physician based upon clinical status, perceived severity of infectious process and/or inability to take antibiotics orally, and appropriateness of sub-acute community dental care. Participants completed a self-administered questionnaire about their medical, dental and social history at the time of enrollment in the OPAT program (Additional file 1). A standardized template was designed and used by the attending infectious diseases physician to document the initial medical assessment and treatment plan (Additional file 2). As part of the study data analysis, antibiotic regimes were categorized as broad or narrow spectrum. Broad-spectrum regimes were defined as consisting of clindamycin alone, ceftriaxone with metronidazole or amoxicillin-clavulanic acid. Narrow-spectrum regimes were defined as consisting of cefazolin, penicillin, amoxicillin, cephalexin or cefadroxil with the potential of concurrent metronidazole.

The total number of patients presenting to acute care facilities with dental infections (including OPAT referred and non-referred) during the study period were obtained from a provincial reporting system according to International Statistical Classification of Diseases and Related Health Problems, 10^th^ Revision, Canada (ICD-10-CA). Given known limitations in the accuracy of ICD code data for dental problems in acute care [21], we used the K04 diagnostic code (diseases of pulp and peri-apical tissues) so as be specific to potential focal infectious processes of non-traumatic origin while not missing cases that may not have been coded using the more specific peri-apical abscess code (K04.4). Total numbers of OPAT referred ADI, study participants and those who declined participation, were obtained from the National Ambulatory Care Reporting System (NACRS) according to ICD-10-CA K04 coding [22]. Healthcare cost estimates were provided through direct communication with Alberta Health Services Billing Information Department (December, 2014) and are reported in Canadian dollars. Cost estimates for hospitalization and OPAT treatment were calculated based on daily operating costs alone: $1580 per day citywide average for non-intensive care medical hospital bed, $288 per day for OPAT treatment.

An area based social deprivation index was used as a proxy of participants’ socio-economic status. The index categorized geographic areas of the city into deprivation quintiles (5^th^ = greatest deprivation, 1^st^ = least deprivation). Derivation and validation of this index by Pampalon et al. is described in detail elsewhere [23, 24]. In brief, 2006 Canadian Census Data was used to determine distribution of deprivation according to social dimensions over geographic zones made up of 400 to 700 individuals constituting the smallest stable census areas. Calgary Zone social deprivation data was provided by the provincial health authority and was matched to participants using primary residence postal code data.

Statistical analysis utilized 2-sided two-sample *t*-tests for comparison of continuous binary variables and linear model based *t*-tests for comparison of continuous variables. Social deprivation quintiles were compared with durations of antibiotics with an *F*-test using a linear model and with binary variables using Chi-squared contingency table test. One-way ANOVA using Scheffe’s method was used for comparisons between antibiotic regimes. Significance was *a priori* defined as an alpha ≤ 0.05 in two-tailed distribution. Data analysis was generated using SAS software, version 9.4 (Cary, NC, USA).

## Results

During the five month-study period 704 individuals presented to regional EDs and urgent care facilities with dental infections of which 343 (49%) severe ADI were referred to the OPAT program. This represented 6% of all OPAT referrals (343/6206) over this period. One hundred and ten individuals referred to OPAT with ADI completed all required documents necessary to be included in the study (32%). Participant characteristics are summarized in Table 1.

**Table 1:** Demographic features of those patient’s referred from acute care to OPAT for ADI management

OPAT ADI (*n* = 110)^a^	*n* (%)
Gender	
Male	50 (45)
Female	60 (55)
Age (years)	
18 – 25	10 [9]
26 – 45	52 (47)
46 – 65	40 [37]
> 65	8 [7]
Participant mean age (SD)	43.9 (14.4)
Dental History	
‘Brush at least daily’ (*n* = 108)	98 (91)
Prior cavities (*n* = 104)	90 (87)
Prior dental infection (*n* = 109)	84 (77)
Prior ADI in the same tooth	39 [36]
‘Have a regular dentist’ (*n* = 108)	87 (81)
Visited a dentist in past 6 months	63 (58)
Dental Insurance (*n* = 107)	70 (65)
Medical History	
Diabetes mellitus (*n* = 108)	7 [6]
Active smoker (*n* = 100)	53 (53) ^e^
Alcohol consumption > 6 beverage/week (*n* = 72)	15 [21]
History of cold sores (*n* = 108)	36 [33]
History of cancer (*n* = 108)	6 [6]
Currently on immunosuppressive^b^ (*n* = 108)	7 [6]
Social Factors	
Employed (*n* = 97)	68 (70)
Social Deprivation Index^c^ − mean (SD) (*n* = 100)	3.3 (1.4)
Number reporting income below LICO^d^ (%) (*n* = 81)	15 [19]

^a^ *n* = 110 unless otherwise specified

^b^Defined as any dosage of oral or inhaled glucocorticoid, chemotherapy, or disease-modifying anti-rheumatic drug

^c^Reported as quintiles - 1 = least deprivation, 5 = most deprivation

^d^LICO = Low Income Cut-Off (‘poverty line’), Statistics Canada defined threshold below which household is expected to spend 20% more than the average household on essential needs [40]

^e^Canadian national smoking prevalence of 16% and Alberta provincial smoking prevalence of 22% (Statistics Canada, 2012)

Clinical data from initial OPAT physician assessment are presented in Table 2. Of those reporting prior ADI involving the same tooth, no association with site of infection (molar vs. non-molar or maxillary vs. mandibular) was present (*p* > 0.1). There was also no association between site of dental infection and the presence of systemic symptoms or having received antibiotics preceding presentation to acute care (*p* > 0.1).

**Table 2:** Participants’ presenting signs and symptoms

OPAT ADI (*n* = 110)^a^	*n* (%)
Duration of Symptoms	
Acute (1–3 days)	37 [34]
Sub-acute (4–14 days)	55 (50)
Chronic (>14 days)	12 [11]
Onset post dental procedure	6 [5]
Systemic Symptoms^b^	33 [30]
Location of Infection – (*n* = 108)	
Mandibular	62 (57)
Molar	44 (41)
Non-molar	18 [17]
Maxillary	46 (43)
Molar	20 [18]
Non-molar	26 [24]

^a^n = 110 unless otherwise specified

^b^Fever, rigors, nausea, vomiting

Antibiotic treatment data is summarized in Table 3. Twenty-two participants (20%) reported completing a course of antibiotics in the preceding three months and 49 (45%) were actively receiving antibiotics for ADI at the time of presentation to acute care. Over the study period a cumulative 1326 antibiotics days were prescribed (415 parenteral, 911 oral). Two participants had antimicrobial therapy discontinued at initial OPAT assessment and 14 (13%) received a single dose of parenteral antibiotics before transitioning to oral treatment. Mean duration of parenteral treatment was significantly longer amongst participants with mandibular focus of infection compared to those with a maxillary focus (4.3 vs. 3.2 day, *p* = 0.01, 95% CI [0.3, 1.9]); however, there was no significant difference in mean total antibiotic (parenteral plus oral) duration when compared by site of infection (*p* > 0.1).

**Table 3:** Antibiotic management of ADI

OPAT ADI (*n* = 110)^a^		
Antibiotic Management		
Received Pre-ED/UC PO antibiotics – *n* (%)	49 (45)	
Days of OPAT IV - median (IQR)	3 [2]	
Days of Post-OPAT PO – median (IQR)	9 [3]	
Total OPAT & Post-OPAT days – median (IQR)	15 [4]	
Antibiotic Regime – *n* (%)		
ED/UC prescription (IV) (*n* = 105)		
Cefazolin & metronidazole^b^	60 (57)	
Clindamycin^c^	22 [21]	
Ceftriaxone & metronidazole^b^	13 [12]	
Other	10 [10]	
OPAT prescription (IV) (*n* = 108)		
Cefazolin & metronidazole^b^	84 (78)	
Ceftriaxone & metronidazole^b^	14 [13]	
Clindamycin^c^	10 [9]	
OPAT discharge prescription (PO) (*n* = 107)		
Cephalexin or cefadroxil both with metronidazole	64 (60)	
Amoxicillin-clavulanic acid	24 [22]	
Clindamycin^c^	16 [15]	
Other	3 [3]	
Regime Specific Duration of Antibiotics – days		
Parenteral (IV)^g^		*p* = 0.2
Narrow spectrum IV regime – mean (SD)^d^	4.2 (2.2)	
Broad spectrum IV regime – mean (SD)^e^	3.2 (1.1)	
Total course (IV & PO) (*n* = 107)		*p* = 0.4
Narrow spectrum regime^f^ – mean (SD)	14.9 (4.3)	
Broad spectrum regime# – mean (SD)	15.2 (5.4)	

*ED* emergency department, *UC* urgent care, *IV* intravenous, *PO* oral route, *IQR* interquartile range, *SD* standard deviation

^a^n = 110 unless otherwise specified

^b^Metronidazole component in either PO or IV formulation

^c^Of those receiving clindamycin 5 had clinical record of a beta-lactam allergy

^d^Narrow spectrum IV regime = cefazolin or penicillin both with metronidazole, PO regime = amoxicillin or cephalexin or cefadroxil with metronidazole

^e^Broad spectrum IV regime = clindamycin alone or ceftriaxone with metronidazole, PO regime = amoxicillin-clavulanic acid or clindamycin

^f^Defined as majority of days of treatment consisting of either broad or narrow spectrum agents

^g^Excluding cases where only a single initial dose of ceftriaxone was given and participants were then discharged on oral antibiotics

Duration of parenteral, oral, and total antibiotic treatments did not significantly differ by duration of symptoms prior to OPAT referral (chronic, sub-acute, acute), presence of systemic symptoms at first OPAT assessment, or report of prior ADI in same tooth (*p* > 0.1). Participant who reported antibiotic treatment preceding presentation to acute care had significantly longer mean total duration of antibiotics compared with those who did not (17.4 vs. 13 days, *p* < 0.001, 95% CI [2.8, 6]) but there was no significant difference in mean duration of parenteral therapy, type of antibiotics used, or the frequency of systemic symptoms between these groups (*p* > 0.1).

Using total duration of antibiotics and duration of parenteral antibiotics as correlates of ADI severity no significant difference was found when compared by having a regular dentist (yes vs. no), dentist visit in past six month, dental insurance status, history of childhood caries or prior dental infection, net household income below low-income cut-off, or age (*p* > 0.1). Average duration of parenteral antibiotics but not total antibiotics was significantly longer amongst female participants compared with males (4.2 vs. 3.3 days, *p* = 0.03, 95% CI [0.9, 2.5]).

Comparing broad versus narrow spectrum antibiotic regimes (defined in Table 3 footnotes), participants who received broad-spectrum parenteral antibiotics had shorter mean duration of parenteral therapy (2.5 vs. 4.2 days, *p* < 0.001, 95% CI [0.8, 2.7]). However when adjusted for those participants that received only a single initial dose of ceftriaxone then discharge from OPAT on oral antibiotics, the mean duration of parenteral therapy was no longer significantly different (3.2 days broad spectrum vs. 4.2 days narrow spectrum, *p* > 0.1). There was no difference between mean total duration (parenteral plus oral) of broad and narrow spectrum regimes (14.9 days narrow spectrum vs. 15.2 days broad spectrum, *p* > 0.1).

The percentage of individuals in each social deprivation index quintile were: Q5 26%, Q4 18%, Q3 8%, Q2 23%, Q1 25% (5^th^ = most deprived, 1^st^ = least deprived). Total duration of antibiotics, duration of parental antibiotics, dentist status, dentist visit in past six month, and dental insurance status were not associated with social deprivation index quintile (*p* > 0.1).

Over the 5-month study period, these 110 study participants received a total of 417 patient days of OPAT care, mean length 3.8 days (SD 2.2), averaging an estimated $1094 cost per course of treatment with a total cumulative cost to the health care system of $120,096. Estimated cost of hospitalization for the equivalent duration of parenteral treatment would have been $717,530.

## Discussion

In the context of a widespread and sustained increases in the numbers of individuals with dental infections presenting to acute medical care, our prospective study of ADI in the OPAT setting provides novel information about the clinical management of patients in a large urban center. Additionally, our study provides contemporary insight into the intersection of the dental and medical healthcare systems in Canada by characterizing the social-medical profile and quantifying the financial and healthcare resource costs of those requiring OPAT care for ADI. The overall severity of dental infections presenting to acute medical care - in our study 49% requiring parenteral antibiotics and OPAT referral - and the related prolonged antibiotic treatment prescribed as management underscore a significant public health problem with implications at the individual, population and health system levels.

Our study’s findings regarding antibiotic treatment on balance suggest no difference between the antibiotic regimes used. Although, when using duration of parenteral therapy as a surrogate of early therapy effectiveness, broader spectrum therapy (ceftriaxone or clindamycin based regimes) appeared to be more effective, shorter mean duration of parenteral treatment, this effect is more likely explained by OPAT practice patterns rather than differential antibiotic efficacy. The practice of giving a single dose of an antibiotic with a longer half-life (i.e. ceftriaxone) to those patients appropriate for oral step down at initial OPAT assessment appears to be common in our OPAT program. When cases that received a single dose of ceftriaxone and were subsequently discharged from OPAT after first assessment were excluded there was no longer a difference in mean duration of parenteral therapy between those who received broad or narrow spectrum regimes. Furthermore, the mean total duration of antibiotic treatment was not significantly different between the various treatment regimes lending support to the use of narrower spectrum, lower toxicity regimes to avoid antibiotic associated complications.

Looking beyond individual management, the cumulative 1326 antibiotic days of treatment amongst the 110 participants in this study represents excessive exposure for a condition that is preventable and for which primary management entails dental intervention [9, 10, 25]. Despite little evidence supporting antibiotic therapy as management of most types of dental infections – a recent systematic review on the topic concluded the quality of available evidence is “very low” [26] - it is estimated that 8 to10% of all primary care antibiotic prescriptions are for treatment of dental infections [27, 28]. Antibiotic prescription patterns for dental infections in the acute care setting are less well quantified. Most acute medical care facilities are not equipped, or do not have dental professionals available, to provide definitive dental care, resulting in temporizing analgesics and antibiotics being the primary management of dental infections in this setting [29]. Highlighting just how common place this approach is, a recent study in the United States found that 56% of emergency room dental visits from a statewide sample received antibiotic prescriptions [30].

The widespread, potentially avoidable, use of antibiotics characterized here and in previous studies on dental infections in general, raises concern about potential significant iatrogenic health risks. At the level of the individual, extended and frequent antibiotic exposures may increase the risk of antibiotic associated diseases such as *Clostridium difficile* colitis [31]. While at the population level such exposures contribute to the rapidly expanding public health emergency of bacterial antibiotic resistance [32, 33]. Our finding that longer total courses of antibiotics were prescribed to individuals who presented to acute care while receiving antibiotics highlights a population amongst whom these risks may be greatest and may benefit most from early triage to dental intervention.

Whereas severe ADI requiring parenteral antibiotics were previously managed with hospitalization, OPAT programs now allow for management in the community [18]. The impacts of this change on antibiotic prescription patterns and the timing of dental interventions have not yet been studied. We hypothesize that unintended consequences of OPAT treatment may be deferral of definitive dental intervention that would typically occur if hospitalized as well as excess durations of parenteral therapy.

As it has been previously reported that patients prefer antibiotic treatment and are often unwilling to accept operative intervention [34], the rapid symptomatic improvement of ADI with parenteral therapy may bolster these preferences and influence convalescent healthcare choices. Our finding that over a third of study participants reported recurrent infections might be a reflection of this. Programmatic features inherent to OPAT management, such as treatment decisions being confined to clinic work hours and standardized multi-dose bag drug formulations used with infusion pumps, may have contributed to longer courses of parenteral therapy than would be used in the hospital setting. The relatively long prescriptions of oral antibiotics (mean of 9 days) seen in our study may in part reflect uncertainty or delays in the timing of dental care follow-up and a desire on the part of the prescribing physician to ensure adequate bridging to definitive management. Together these issues highlight a need for improved pathways to dental care along with patient and physician education around optimal dental infection management. The infectious diseases expertise of OPAT teams should facilitate enhanced antimicrobial stewardship, however our finding suggests practices in this regard could be improved. Collaboration with existing hospital and regional antimicrobial stewardship programs and implementation of checklists to aid in OPAT service decisions have been proposed elsewhere [35] and could further reduce risks associated with ADI antibiotic management.

Our cost saving findings must be interpreted with caution as they only capture direct cost estimates of current medical management practices missing the indirect costs associated with excess antibiotic use. In-line with previous OPAT cost-effectiveness research [36], our conservative estimate of $597,434 (CAN) (83%) cost savings compared with hospitalization for the full course of parenteral antibiotic treatment suggests a role for OPAT programs in ADI management. However, as OPAT therapy only serves as a bridge to definitive dental care these cost-savings may only be short term if not complimented by timely engagement with dental services. Further research evaluating the comparative cost-effectiveness of OPAT treatment compared with primary prevention and hospital-based dental interventions is needed to better understand total costs and inform dental health policy reform.

The socioeconomic profile of participants presenting to EDs with severe ADI in our study and high rates of reported dental insurance coverage and connection to dental care without apparent association to social deprivation are in contrast to findings from previous retrospective studies on dental infections in acute medical care [11, 12, 37]. In further contrast, a social gradient has been observed regionally in Alberta where, between 2007 and 2013, residents in the lowest income group were twice as likely compared with the highest income group to seek dental care in emergency departments (13 visits per 1,000 vs. 6.2 visits per 1,000) [38]. Although our findings may reflect true demographic differences between the sub-group of severe ADI evaluated in our study compared to the more inclusive dental infection populations in previous retrospective studies, both selection and information bias may have also influenced our findings.

Our study has a number of notable limitations. Most importantly, we captured only a severe subset of ADIs, those presenting to EDs and felt to require parenteral antibiotics. How those patients with severe ADIs differ from the general population experiencing any ADIs cannot be established based on our referral base. Furthermore, with only a third of eligible OPAT referrals participating in the study our sample may not be representative of the source population. Our use of written consent forms and questionnaires may have limited participation of those illiterate or non-English speaking while questions about sensitive medical and social information may have lead to subject reporting bias. Use of objective postal code reporting and assigning a deprivation index accordingly was intended to mitigate such reporting bias. However, information bias may have resulted from the social deprivation index itself as it was based upon census data from 2006 since which time Calgary has undergone considerable economic growth resulting in potential shifts in the socioeconomic composition of communities. Finally, our study did not capture those patients admitted to hospital with ADI who may have disproportionately represented lower socioeconomic groups. With regard to our finding of high rates of reported dental insurance coverage and connection to dental care, previous research has noted that this does not necessarily translate into regular access to dental care as financial barriers, such as excessive deductibles, and an individual’s resistance to dental intervention may still remain [15, 30, 39].

The epidemic of dental infections seeking acute medical care represents a growing public health problem in North America. Beyond the well-established healthcare costs of this epidemic, related extensive antibiotic usage poses an additional public health threat by way of potentially contributing to bacterial antibiotic resistance. Addressing these interconnected issues as they relate to ADI demands improved integration of medical and dental services to ensure timely definitive management. While OPAT programs may offer short-term cost-efficiency compared to hospitalization for the medical management of a selected subset of severe ADI, without improved community pathways to definitive dental care and adherence to antimicrobial stewardship best practices these saving may be outweighed by unintended delays in definitive management and risks associated with excessive antibiotic exposures.

## Additional file

Additional file 1: Self-administered participant questionnaire (attached as separate file) (DOCX 103 kb)
Additional file 2: OPAT ADI initial medical assessment template (attached as separate file) (DOCX 236 kb)

## Abbreviations

ADI: Acute Dental Infections
ED: Emergency Department
OPAT: Outpatient Parenteral Antibiotic Therapy
