# Supplementary material for: Acute dental infections managed in an outpatient parenteral antibiotic program setting: prospective analysis and public health implications
Source: BMC Infect Dis. 2017 Mar 9;17:202. doi: 10.1186/s12879-017-2303-2 (PMC5345191; doi:10.1186/s12879-017-2303-2)
Supplement: Additional file 1: — Self-administered participant questionnaire (attached as separate file) (DOCX 103 kb) [file 12879_2017_2303_MOESM1_ESM.docx]

**Participant Questionnaire**

Full Name: ___________________________ Phone 1: _________________________________

Phone 2: _________________________________

Time of day that you prefer to be contacted:_______________________________________________

Email Address (if preferred means of contact): ____________________________________________

**Dental History:**

Do you currently have a dental care provider (dentist)? YES / NO (circle one)

If YES, have you seen your dentist in the last 6months? YES / NO

Do you currently have dental insurance? YES / NO (Example: Alberta Blue Cross, Great West Life)

Do you brush your teeth daily? YES / NO

Did you have cavities (dental caries) as a child? YES / NO

Have you previously had treatment for a dental infection (cavities, root canal…)? YES / NO

If YES, 1) is your current problem a recurrence: YES / NO

2) List type of problems, any complication and the year of occurrence:__________________

__________________________________________________________________________

__________________________________________________________________________

**Medical Issues:**

Do you have diabetes? YES / NO

Do you have a medical condition that compromises your immune system? YES / NO

If YES, please list: __________________________________________________

Are you taking any immunosuppressive medications (ie/ inhaled or oral steroids)? YES / NO

Do you have a history of cancer (excluding skin cancer)? YES / NO

Have you previously had ‘cold sores’ (oral herpes virus infection)? YES / NO

Did you receive any antibiotics in the 6-month period before your dental infection? YES / NO

**Social Habits:**

Do you smoke or chew tobacco? YES / NO

Do you drink alcohol? YES / NO

If YES, how many alcoholic beverages per week: ______

Are you currently employed, working? YES / NO

What is your net monthly household income (before taxes)? (see below for details on how to estimate)

_____________________________________________________________________________

Estimating monthly household income:

| **Your income per month** | **Your spouse / partner’s income per month** | **Income per month of other family members living in your household**  (18 years and older) | **Combined Monthly Household Income** |
| --- | --- | --- | --- |
|  | **+** | **+** | **= $** |
