# Supplementary material for: Acute dental infections managed in an outpatient parenteral antibiotic program setting: prospective analysis and public health implications
Source: BMC Infect Dis. 2017 Mar 9;17:202. doi: 10.1186/s12879-017-2303-2 (PMC5345191; doi:10.1186/s12879-017-2303-2)
Supplement: Additional file 2: — OPAT ADI initial medical assessment template (attached as separate file) (DOCX 236 kb) [file 12879_2017_2303_MOESM2_ESM.docx]

DENTAL INFECTION STUDY MD TEMPLATE

**ID:**

**HPI:**

**Duration:** acute [1-3 day] ☐, sub-acute [4-14 day] ☐, chronic [>14 day] ☐, Post trauma/OR ☐

**Local Symptoms:** dyspnea/wheeze ☐, dysphagia ☐, odynophagia ☐, ocular ☐, auditory ☐

**Systemic Symptoms:** No ☐, Yes ☐ → fever ☐, rigors ☐, nausea ☐, anorexia ☐, diarrhea ☐

**Pre HPTP Care:**

- Antibiotics:

- Interventions:

**Medical Profile:**

Medical History (Oral ☐ Renal ☐ Cardiac ☐ Immune Disorder ☐ DM ☐):

Dental History: (prior infections ☐ prior surgery ☐ [record details as free text])

**Allergies:**

**Social Factors:** (Smoking ☐, Alcohol ☐, IVDU ☐, Other recreational drugs ☐):

Has Primary Dentist: Yes ☐, No ☐

Has Dental Insurance: Yes ☐, No ☐

**Assessment:** (MARK DENTAL DIAGRAM ON BACK)

(fever ☐, stridor ☐, trismus[<2cm opening]☐, cranial palsy ☐) other:

Date: HPTP Site:

Patient Label

(add free text as needed, 2 sided)

DENTAL INFECTION STUDY MD TEMPLATE

Patient Label

**Mark affected teeth/area(s) w/ ‘X’** **Notes:**


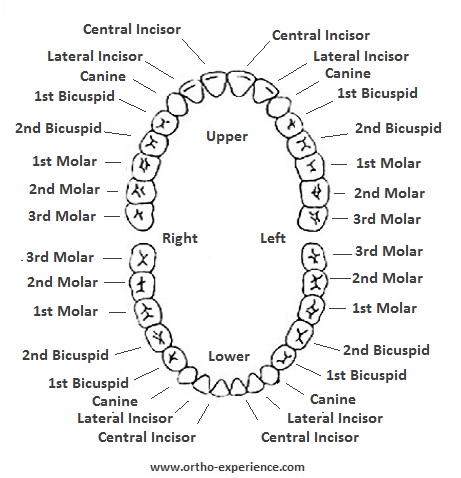


**IMPRESSION:**

**PLAN (initial therapy):**

☐ Antibiotics: PO IV

☐ Amox–Clav 875 mg PO BID x ___ ☐ Cefazolin 1 -2 g IV Q8H x ___

☐ Clinda 300-450 mg PO QID x ___ ☐ Flagyl 500mg IV Q8-12H x ___

☐ Flagyl 500 mg PO BID x ___ ☐ Pen G 4mil U IV q6 x ______

☐ Keflex 500 mg PO QID x ___ ☐ Ceftriaxone 1-2g IV Q24 x ___

☐ Other:

☐ Analgesics:

☐ Blood Work:

☐ Imaging: Dental XR☐, CT H&N ☐, CT Orbits ☐, MRI ☐, Nuc Med Study ☐

☐ Referral: ENT☐, OMF Dental ☐, Social Work ☐, Other ☐ =

☐ Follow-up:

Date: HPTP Site:
